# Estimates of Serial Interval and Reproduction Number of Sudan Virus, Uganda, August–November 2022

**DOI:** 10.3201/eid2907.221718

**Published:** 2023-07

**Authors:** Valentina Marziano, Giorgio Guzzetta, Ira Longini, Stefano Merler

**Affiliations:** Bruno Kessler Foundation, Center for Health Emergencies, Trento, Italy (V. Marziano, G. Guzzetta, S. Merler);; University of Florida, Colleges of Public Health and Health Professions and Medicine, Gainesville, Florida, USA (I. Longini)

**Keywords:** Sudan virus, viruses, serial interval, reproduction number, Ebola, Uganda

## Abstract

We estimated the mean serial interval for Sudan virus in Uganda to be 11.7 days (95 CI% 8.2–15.8 days). Estimates for the 2022 outbreak indicate a mean basic reproduction number of 2.4–2.7 (95% CI 1.7–3.5). Estimated net reproduction numbers across districts suggest a marked spatial heterogeneity.

On September 20, 2022, Uganda’s Ministry of Health declared an Ebola disease outbreak after a case caused by Sudan virus (SUDV) was confirmed in a village in Mubende District (*1*). Suspicious deaths in the same district had occurred earlier in the month. Investigations conducted by the National Rapid Response Team allowed the identification of probable SUDV cases dating back to mid-August 2022 (*2*,*3*). As of November 2, 2022, a total of 149 cases (131 PCR-confirmed and 18 probable) were reported in the country; most cases occurred in the districts of Mubende (63 confirmed, 17 probable), Kassanda (42 confirmed, 1 probable), and Kampala (18 confirmed) (*2*) (Figure 1, panels A–C). On January 11, 2023, the outbreak was declared over with a total of 164 cases (142 confirmed, 22 probable) (*4*).

**Figure 1:**
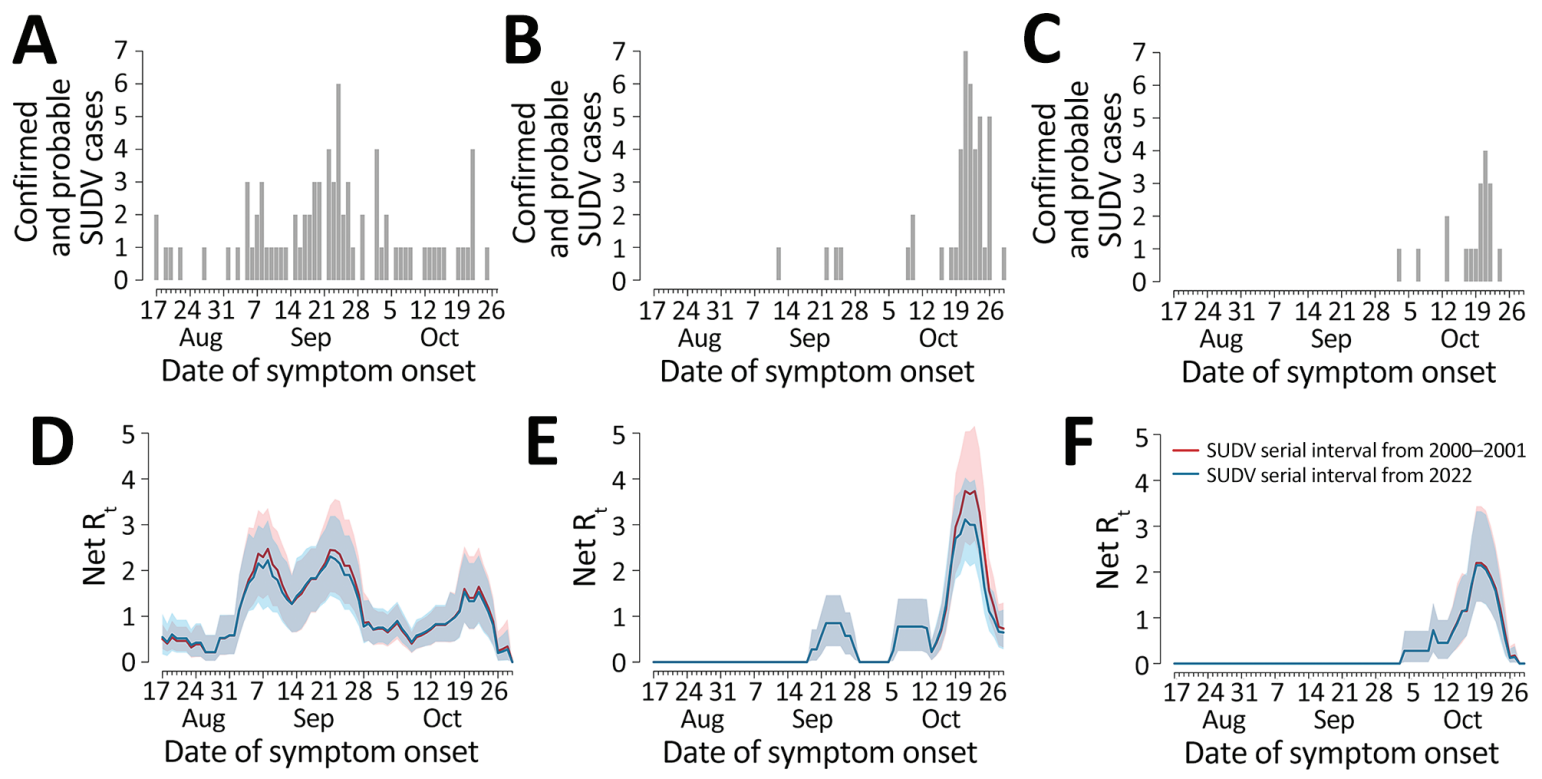
Epidemic curves and reproduction numbers for SUDV outbreaks in Mubende, Kassanda, and Kampala districts, Uganda, August–November 2022. A–C) Number of confirmed and probable cases by date of symptom onset in the 3 considered districts (comprising 95% of cases reported in the whole country, as of November 2, 2022) (*2*): Mubende (A), Kassanda (B), and Kampala (C). D–F). Estimates of the net reproduction number over time in the 3 districts: Mubende (D), Kassanda (E), and Kampala (F). Estimated from the corresponding epidemic curves by date of symptom onset were computed using the serial interval distributions from the 2000–2001 outbreak (*5*) (red) and the 2022 outbreak (*6*) (blue). Shaded areas represent 95% CIs of estimates. We assumed that the first case of the epidemic curve in each district was imported and that all the others were locally transmitted. SUDV, Sudan virus.

## The Study

We provide 2 estimates of the serial interval distribution (the time elapsed between the symptom onset in an index case-patient and in their secondary case-patients) by using observed serial intervals in infector–infectee pairs as identified during contact-tracing operations conducted in 2 SUDV outbreaks in Uganda, during 2000–2001 (24 pairs) (*5*) and the 2022 outbreak (12 pairs) (*6*). We fitted 3 families of distributions (Weibull, Gamma, and log-normal) with a possible offset (*5*,*6*). We obtained the best fit for the serial interval distribution for both datasets with a Weibull distribution. We estimated the mean serial interval to be 12.0 days (95% CI 10.0–14.2 days) by using the 2000–2001 outbreak data and 11.7 days (95% CI 8.2–15.8 days) by using the 2022 outbreak data.

We then used estimates of the serial interval as a proxy of the generation time to compute the basic (R_0_) and net (R_t_) reproduction numbers. We defined R_0_ as the average number of secondary infections generated by an infectious person in a fully susceptible population. If R_0_ <1, transmission is expected to fade out, whereas if R_0_ >1, the epidemic has the potential to continue; the larger R_0_, the more difficult it is to control the epidemic. We defined R_t_ as the average number of secondary cases per infectious person at time t; R_t_ is key to monitor the effectiveness of interventions throughout the epidemic. In the main analysis, we computed R_t_ and R_0_ by using a method based on the renewal equation in the formulation by Cori et al. (*7*). In additional analyses, we used the assumption of exponential (*8*) or subexponential (*9*) growth of the cumulative case incidence curve to compute R_0_. We also provided an alternative estimate of R_t_ obtained by applying a recently proposed approach (*10*) that has been suggested to perform better with low case counts (Appendix).

R_0_ of the 2022 outbreak, as estimated from the epidemic curve of Mubende District, was 2.7 (95% CI 1.9–3.5) based on the serial interval distribution from the 2000–2001 outbreak. R_0_ was 2.4 (95% CI 1.7–3.3) based on the serial interval distribution from the 2022 outbreak.

We estimated R_t_ in the 3 districts and according to the 2 estimated serial interval distributions (Figure 1, panels D–F). For convenience and given their similar values, numbers reported hereafter refer to the 2000–2001 serial interval; corresponding numbers for the 2022 serial interval are reported separately (Appendix). In Mubende District, R_t_ reached a peak during September 21–23, 2022, with an estimated value for R_t_ that was close to R_0_ (mean 2.4 [95% CI 1.5–3.5]). R_t_ fell rapidly below the epidemic threshold during September 28–October 15 (mean 0.71 [95% CI 0.50–0.91]), possibly because of control interventions and population behavior changes after awareness of the outbreak had increased. In the second half of October, R_t_ increased again, reaching a peak of 1.34 (95% CI 0.78–2.13) in the week October 18–24. In the districts of Kassanda and Kampala, R_t_ increased rapidly in the second half of October. In Kassanda, R_t_ reached a peak of 3.5 (95% CI 2.5–4.9) during October 20–24. In Kampala, the peak R_t_ value was 2.0 (95% CI 1.3–3.2) during October 18–22.

**Figure 2:**
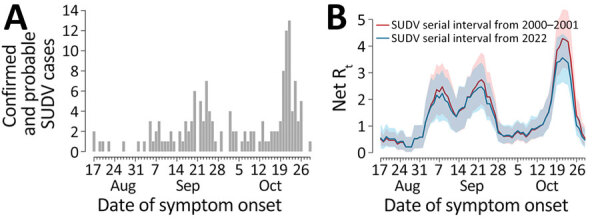
Epidemic curves and reproduction number for SUDV outbreaks, Uganda, August–November 2022. A) Number of confirmed and probable cases, by date of symptom onset, in Uganda as obtained from district-level epidemic curves reported previously (*2*). B) Net reproduction number over time in Uganda, as estimated from the epidemic curve, by date of symptom onset, using the serial interval distributions from the 2000–2001 outbreak (*5*) (red) and from the 2022 outbreak (*6*) (blue). Shaded areas represent 95% CIs of estimates. We assumed that the first case of the epidemic curve was imported and that all the others are locally transmitted. SUDV, Sudan virus.

Estimates of R_t_ at the national level (Figure 2) are characterized by 2 peaks in September, which were driven by SUDV transmission in Mubende. A third marked peak that occurred in the second half of October was sustained mainly by increasing transmission in the districts of Kassanda and Kampala, as well as by a resurgence in Mubende.

## Conclusions

We estimated the distribution of the serial intervals for SUDV by using 2 different datasets from the 2000–2001 outbreak and from the ongoing outbreak in Uganda, finding similar distributions and an average serial interval of ≈12 days. On the basis of those estimates and publicly available data on the epidemic curve made available by the Ugandan Ministry of Health (*2*), we found the R_0_ in Mubende District, the district first and most affected by the current outbreak, was ≈2.4–2.7, although with broad uncertainty (95% CI 1.7–3.5). Those estimates are in line with previous estimates for SUDV, which ranged from 1.3 to 4.1 (*11*), and with estimates obtained using alternative methods (*8*,*9*) (Appendix). After a temporary containment of the outbreak from the end of September until mid-October 2022, with R_t_ hovering around 0.7, the third week of October marked a resurgence of transmissibility in Mubende (R_t_ ≈1.3) and the emergence of new outbreaks in the Kassanda (R_t_ ≈3.5) and Kampala (R_t_ ≈2.0) districts. The R_0_ associated with the national aggregation shows the same temporal features but suggests even higher numbers for the R_0_ in the fourth week of October (mean R_0_ ≈4), demonstrating the important role played by spatial heterogeneity of transmission in the 2022 outbreak.

Our estimates should be interpreted with caution, considering the following limitations. Estimates of the serial interval distributions are based on small numbers of infector–infectee pairs. Reproduction numbers have a broad uncertainty because of limited case numbers and may be substantially affected by superspreading events, biasing estimate upward with respect to the average transmissibility in the general population. However, the proposed estimates are in line with those obtained using an alternative method that was suggested to be more robust for low case counts (*10*) (Appendix). Moreover, a potential increase in reporting rates of confirmed cases after the discovery of the first cases in each district may inflate the estimate of the reproduction numbers.

Given the geographic expansion of the outbreak, which included urban settings, and the absence of therapeutics and licensed vaccines to treat and prevent SUDV, by the end of October 2022, the World Health Organization assessed the risk for infection at the national level to be very high (*12*). However, the rapid deployment of interventions (including contact tracing, isolation of case-patients, and informational activities to promote community engagement) was sufficient to contain the outbreak, which was declared over on January 11, 2023. Our analysis provides quantitative information on the evolution of SUDV transmissibility in the different districts of Uganda during the 2022 outbreak. Estimates provided for the serial interval may be instrumental in planning control interventions in possible future outbreaks of SUDV.

Appendix Additional information on estimates of serial interval and reproduction number of Sudan virus, Uganda, August–November 2022.
