# Supplementary material for: Estimates of Serial Interval and Reproduction Number of Sudan Virus, Uganda, August–November 2022
Source: Emerg Infect Dis. 2023 Jul;29(7):1429–32. doi: 10.3201/eid2907.221718 (PMC10310358; doi:10.3201/eid2907.221718)
Supplement: Appendix — Additional information on estimates of serial interval and reproduction number of Sudan virus, Uganda, August–November 2022. [file 22-1718-Techapp-s1.pdf]

Article DOI: https://doi.org/10.3201/eid2907.221718

Estimates of Serial Interval and Reproduction Number of Sudan Virus, Uganda, August–November 2022

Appendix

Serial interval

The serial interval is defined as the difference between the date of symptom onset of a case and those of his secondary cases. We estimated the serial interval distribution of Sudan Virus (SUDV) using two different datasets:

- previously published data on SUDV transmission chains from the 2000–2001 outbreak in Uganda (*1*);
- data reported by the Ministry of Health of Uganda on the transmission chains generated by one case in the 2022 SUDV outbreak in Uganda (*2*).

For each dataset, we considered observed serial intervals from infector–infectee pairs for which the symptom onset date was known (24 infector–infectee pairs from (*1*); 12 infector–infectee pairs from (*2*)) and we fitted three families of distributions to the data (Weibull, Gamma, log-normal) allowing for an offset to reproduce the observation that no serial interval was below 4 and 2 days respectively in the considered data (*1*,*2*).

For both datasets, the best fitting distribution according to maximum likelihood, Akaike Information Criterion and Bayesian information Criterion was the Weibull distribution (Appendix Table 1).

The resulting estimates of the parameters of the two Weibull distributions of the serial intervals are reported in Appendix Table 2. The best-fitting cumulative density functions estimated for the serial intervals are shown in Appendix Figure 1 along with the cumulative distribution of observed serial intervals from the two datasets.

For the computation of the net reproduction number R_t and the basic reproduction number R_0, we assume that the distribution of the generation time (i.e., the difference between the date of infection of a case and those of his secondary cases) can be approximated by the distribution of the serial interval.

Basic reproduction number R_0 and net reproduction number R_t

The basic reproduction number R_0 is defined as the average number of secondary infections generated by an infectious individual in a fully susceptible population. R_0 is a key epidemiologic parameter characterizing the transmission potential of an infectious pathogen in the early epidemic phase. If $R_0 < 1$, transmission is expected to fade out even in absence of control, whereas if $R_0 > 1$, the epidemic has the potential to continue; the larger R_0, the more difficult it is to control the epidemic.

During outbreaks, the transmissibility of an infectious pathogen may vary, e.g., because of population immunity, changes in social contacts of the population, or the implementation of control measures. Temporal variations in the transmission potential are monitored through the net reproduction number, R_t, defined as the average number of secondary cases per infectious individual at time t. Both parameters play an essential role in the planning and design of control measures, as well as in the monitoring of their effectiveness.

Different methods have been proposed in the literature to estimate the net reproduction number R_t from case incidence data (*3–5*). In this study, we estimate the distribution of the net reproduction number R_t at the national and district level by applying the statistical method of Cori et al. (*3*), based on the knowledge of the distribution of the generation time and on the time series of cases.

The posterior distribution of R_t for any time point t was estimated by applying the Metropolis-Hastings MCMC sampling to a likelihood function defined as follows:

$$\mathcal{L} = \prod_{t=1}^{T} P\left(C(t);\ R_t \sum_{s=1}^{t} \varphi(s) C(t-s) \right)$$

Where:

- $P(k; \lambda)$ is the probability mass function of a Poisson distribution (i.e., the probability of observing k events if these events occur with rate λ).
- $C(t)$ is the daily number of new cases having symptom onset at time t;
- R_t is the net reproduction number at time t to be estimated;
- $\varphi(s)$ is the integral of the probability density function of the generation time evaluated between day s-1 and s; we considered the distribution of the serial interval estimated above as an approximation of the distribution of the generation time.

We considered only the case with earliest symptom onset as imported case.

For comparison, we also computed estimates of R_t using the method proposed by Parag (*5*).

To estimate the basic reproduction number, in the main analysis, we use the method for R_t proposed by Cori et al. based on the renewal equation, forcing the value of R_t to be constant and equal to the value of R_0 between September 20 and 24. The time window is chosen in such a way that the impact of population behavior change, control interventions and accumulation of immunity can be considered negligible and that a sufficient number of cases have had symptom onset. The first case in the outbreak was confirmed on September 20, 2022.

We also consider alternative methods to estimate the basic reproduction number R_0. If the cumulative case incidence in the early phase of the epidemic is assumed to grow exponentially, R_0 can be estimated by fitting the exponential growth rate r (*6*,*7*). If the growth of cumulative cases is sub-exponential, a generalized-growth model may be more appropriate where the growth rate r is estimated in combination with an additional parameter p representing the deceleration of growth (*8*,*9*).

We provide estimates of R_0 for the Mubende district from both approaches estimating their parameters through nonlinear least-square fitting to the cumulative case incidence in the first 36 days (about three generations of cases), from August 18 to September 22, 2022.

Additional results

Alternative estimates of the basic reproduction number

The fit of the cumulative case incidence using the exponential growth model and the generalized-growth model is reported in Appendix Figure 2. Using the exponential growth model (*6*,*7*), we estimated an exponential growth rate $r = 0.082$ (95% CI: 0.080- 0.084) and a corresponding basic reproduction number $R_0 = 2.68$ (95% CI: 2.62–2.74). Using the generalized-growth model (*8*,*9*), we estimated a growth rate $r = 0.11$ (95% CI: 0.05–0.19) and a growth deceleration parameter p = 0.84 (95% CI: 0.64–1.15), resulting in a reproduction number $R_g = 1.99$ (95% CI: 1.40–2.90). Both results are in line with those of the main analysis (mean 2.4–2.7, range of 95% CIs: 1.7–3.5).

Estimates of R_t considering the 2022 serial interval

We report hereafter estimates of the net reproduction numbers over time as obtained by assuming the 2022 serial interval (*2*) (blue lines in Figure 1 and 2 of the main text).

In Mubende district, the net reproduction number R_t reached a peak between September 21 and September 23, 2022, with an estimated value for R_t that was close to R_0 (mean September 21–23: 2.2; 95% CI: 1.4–3.1). The reproduction number fell rapidly below the epidemic threshold between September 28 and October 15 (mean September 28 - October 15: 0.72, 95% CI: 0.54–0.93), possibly due to implemented control interventions and population behavior change following increased awareness of the outbreak. In the second half of October the net reproduction number increased again reaching a peak of 1.32 (95% CI: 0.75–2.03) in the week October 18–24. In the district of Kassanda and Kampala, the net reproduction number increased rapidly in the second half of October. In Kassanda, it reached a peak of 2.9 (95% CI: 2.1–3.8) between October 20 and October 24. In Kampala, the peak value was 2.0 (95% CI: 1.3–3.1) between October 18 and 22.

Estimates of R_t and R_0 are obtained using the serial interval as a proxy of the generation time. Given the relationship of direct proportionality existing between the reproduction number and the generation time (*6*,*7*), estimates of the reproduction numbers obtained using the 2000–2001 serial interval (mean: 12 days) are slightly higher than those obtained using the 2022 serial interval (mean: 11.7 days).

Estimates of R_t obtained using the *Epifilter* method

Finally, we estimated R_t for the district of Mubende using the method proposed in (*5*) (R implementation of *EpiFilter* available at https://github.com/kpzoo/ EpiFilter). We applied *Epifilter* assuming a uniform prior distribution for R_t over a grid of size m = 1000, defined between R_{min} = 0.01 and R_{max} = 10, and a state noise parameter η = 0.1. Except for the first 10 days, when the cumulative number of observed cases was still very low (6), the 95% CI of our estimates always intersect with the 95% CI of estimates obtained through this alternative method (Appendix Figure 3).

References

1. Francesconi P, Yoti Z, Declich S, Onek PA, Fabiani M, Olango J, et al. Ebola hemorrhagic fever transmission and risk factors of contacts, Uganda. Emerg Infect Dis. 2003;9:1430–7. PubMed https://doi.org/10.3201/eid0911.030339

2. The Republic of Uganda Ministry of Health. Press statement. Update on the Ebola virus disease outbreak and containment measures. 26 October, 2022 [cited 2023 Jun 6]. https://www.health.go.ug/cause/update-on-the-ebola-virus-disease-outbreak-and-containment-measures

3. Cori A, Ferguson NM, Fraser C, Cauchemez S. A new framework and software to estimate time-varying reproduction numbers during epidemics. Am J Epidemiol. 2013;178:1505–12. PubMed https://doi.org/10.1093/aje/kwt133

4. Wallinga J, Teunis P. Different epidemic curves for severe acute respiratory syndrome reveal similar impacts of control measures. Am J Epidemiol. 2004;160:509–16. PubMed https://doi.org/10.1093/aje/kwh255

5. Parag KV. Improved estimation of time-varying reproduction numbers at low case incidence and between epidemic waves. PLOS Comput Biol. 2021;17:e1009347. PubMed https://doi.org/10.1371/journal.pcbi.1009347

6. Chowell G, Hengartner NW, Castillo-Chavez C, Fenimore PW, Hyman JM. The basic reproductive number of Ebola and the effects of public health measures: the cases of Congo and Uganda. J Theor Biol. 2004;229:119–26. PubMed https://doi.org/10.1016/j.jtbi.2004.03.006

7. Wallinga J, Lipsitch M. How generation intervals shape the relationship between growth rates and reproductive numbers. Proc Biol Sci. 2007;274:599–604. PubMed https://doi.org/10.1098/rspb.2006.3754

8. Chowell G, Viboud C, Simonsen L, Moghadas SM. Characterizing the reproduction number of epidemics with early subexponential growth dynamics. J R Soc Interface. 2016;13:20160659. PubMed https://doi.org/10.1098/rsif.2016.0659

9. Viboud C, Simonsen L, Chowell G. A generalized-growth model to characterize the early ascending phase of infectious disease outbreaks. Epidemics. 2016;15:27–37. PubMed https://doi.org/10.1016/j.epidem.2016.01.002

10. Republic of Uganda Ministry of Health, World Health Organization–Uganda. Ebola virus disease—situation report 42 [cited 2023 Jun 6]. https://www.afro.who.int/sites/default/files/2022-11/Ug_EVD_SitRep%2342.pdf

Appendix Table 1. Fit of the SUDV serial interval. Log-likelihood, Akaike Information Criterion (AIC) score and Bayesian information criterion (BIC) score as obtained from the fit of the three families of distributions to the two different datasets (*1*,*2*). The best-fitting value of the three scores is highlighted in bold.

	2000–2001 SUDV outbreak (*1*) (n = 24)			2022 SUDV outbreak (*2*) (n = 12)		
Distribution	Log-likelihood	AIC	BIC	Log-likelihood	AIC	BIC
Gamma	−73.3	150.7	153.1	−39.6	83.3	84.2
Weibull	−72.4	148.9	151.2	−39.0	82.1	83.1
Log-normal	−75.8	155.6	158	−41.2	86.3	87.3

Appendix Table 2. Parameters of the Weibull distribution of the serial interval as estimated from the different datasets.

Dataset	2000–2001 SUDV outbreak (*1*) (n = 24)	2022 SUDV outbreak (*2*) (n = 12)
Offset (days)	3	1
Shape (mean)	1.76 (1.35–2.60)	1.63 (1.15–3.04)
Scale (95% CI)	10.14 (7.77–12.58)	11.97 (7.9–16.56)
Mean (95% CI) (days)	12 (10–14.2)	11.7 (8.2–15.8)

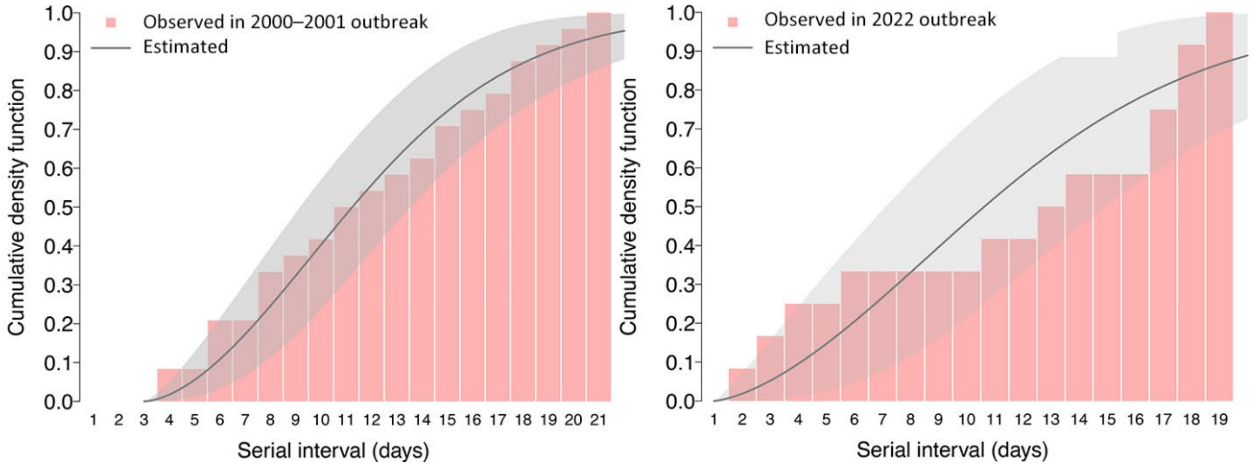

Appendix Figure 1. A) Cumulative density function of the serial interval of SUDV as estimated from 24 infector–infectee couples with known symptom onset in the 2000–2001 outbreak in Uganda (*1*) (mean, solid line; 95% CI, shaded areas). Bars represent the cumulative distribution of the serial interval observed for the considered couples. B) As A but as estimated from 12 infector–infectee couples with known symptom onset in the 2022 SUDV outbreak in Uganda (*2*).

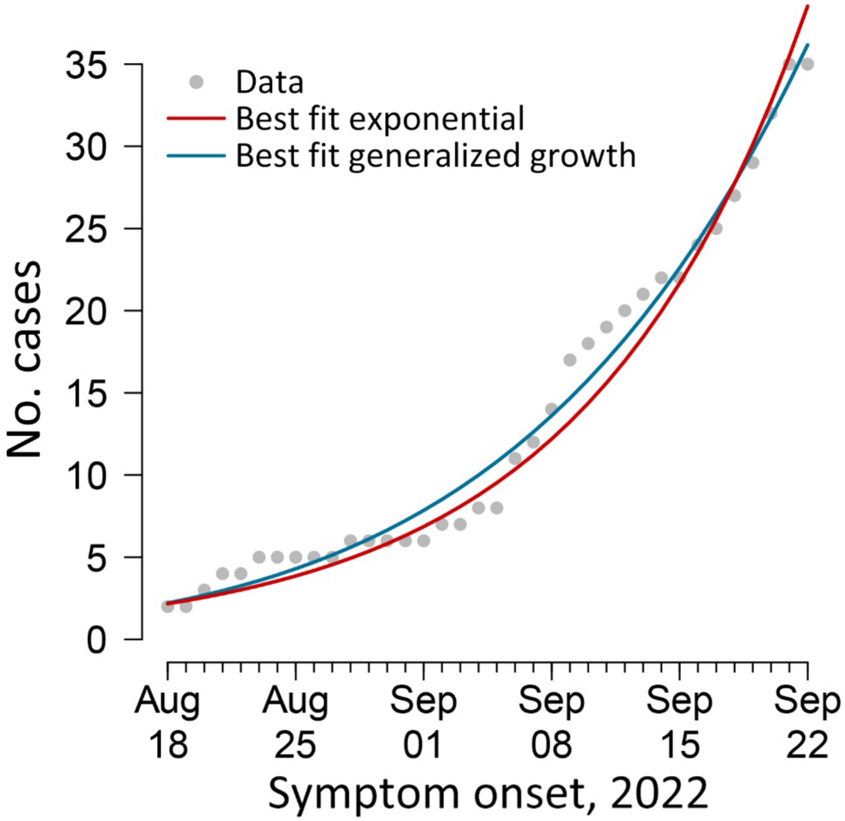

Appendix Figure 2. Cumulative case incidence in the first 36 days (about three generations of cases), from August 18 to September 22, 2022, in the district of Mubende: data (gray) (*10*); fit obtained with the exponential growth model (*6*,*7*) (red); fit obtained with the generalized-growth model (*8*,*9*) (blue).

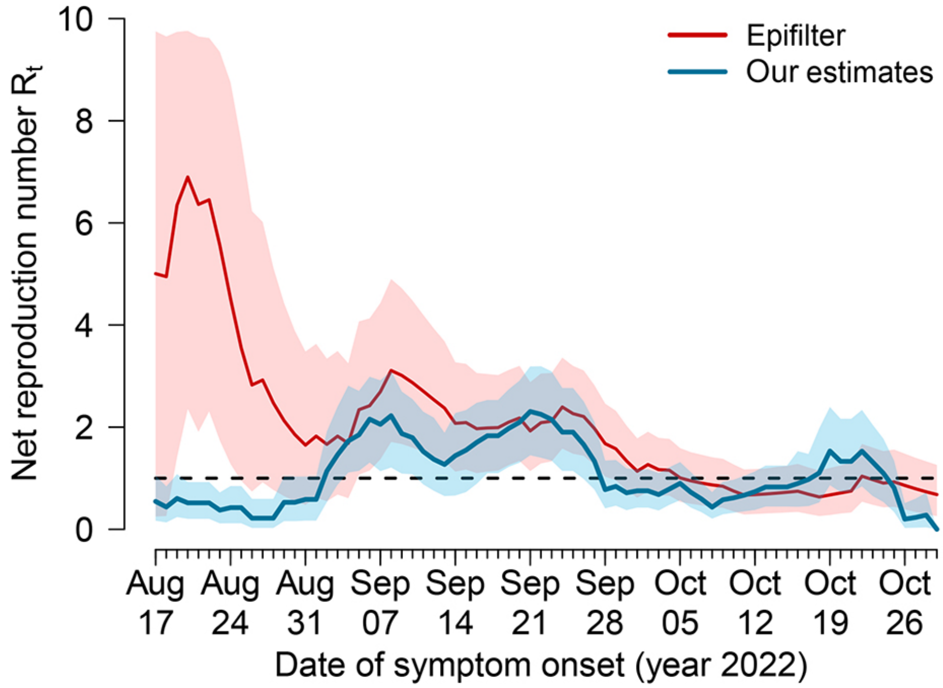

Appendix Figure 3. Net reproduction number over time (R_t) in the district of Mubende, as estimated from the epidemic curve by date of symptom onset and using the serial interval distributions from 2022 outbreak (*2*): method based on Cori et al. (*3*) (blue); *Epifilter* method (*5*) (red). Shaded areas represent 95% CI of estimates.
